# A novel collaborative filtering model for LncRNA-disease association prediction based on the Naïve Bayesian classifier

**DOI:** 10.1186/s12859-019-2985-0

**Published:** 2019-07-17

**Authors:** Jingwen Yu, Zhanwei Xuan, Xiang Feng, Quan Zou, Lei Wang

**Affiliations:** 1 College of Computer Engineering & Applied Mathematics, Changsha University, Changsha, Hunan People’s Republic of China; 2 Key Laboratory of Intelligent Computing & Information Processing, Xiangtan University, XiangTan, People’s Republic of China; 3 Institute of Fundamental and Frontier Sciences, University of Electronic Science and Technology of China, Chengdu, People’s Republic of China; 4 School of Computer Science and Technology, Tianjin University, Tianjin, People’s Republic of China

**Keywords:** lncRNA-disease associations, Original tripartite network, Item-based collaborative filtering, Updated tripartite network, naïve Bayesian classifier

## Abstract

**Background:**

Since the number of known lncRNA-disease associations verified by biological experiments is quite limited, it has been a challenging task to uncover human disease-related lncRNAs in recent years. Moreover, considering the fact that biological experiments are very expensive and time-consuming, it is important to develop efficient computational models to discover potential lncRNA-disease associations.

**Results:**

In this manuscript, a novel Collaborative Filtering model called CFNBC for inferring potential lncRNA-disease associations is proposed based on Naïve Bayesian Classifier. In CFNBC, an original lncRNA-miRNA-disease tripartite network is constructed first by integrating known miRNA-lncRNA associations, miRNA-disease associations and lncRNA-disease associations, and then, an updated lncRNA-miRNA-disease tripartite network is further constructed through applying the item-based collaborative filtering algorithm on the original tripartite network. Finally, based on the updated tripartite network, a novel approach based on the Naïve Bayesian Classifier is proposed to predict potential associations between lncRNAs and diseases. The novelty of CFNBC lies in the construction of the updated lncRNA-miRNA-disease tripartite network and the introduction of the item-based collaborative filtering algorithm and Naïve Bayesian Classifier, which guarantee that CFNBC can be applied to predict potential lncRNA-disease associations efficiently without entirely relying on known miRNA-disease associations. Simulation results show that CFNBC can achieve a reliable AUC of 0.8576 in the Leave-One-Out Cross Validation (LOOCV), which is considerably better than previous state-of-the-art results. Moreover, case studies of glioma, colorectal cancer and gastric cancer demonstrate the excellent prediction performance of CFNBC as well.

**Conclusions:**

According to simulation results, due to the satisfactory prediction performance, CFNBC may be an excellent addition to biomedical researches in the future.

## Background

Recently, accumulating evidences have indicated that lncRNAs (Long non-coding RNAs) are involved in almost the entire cell life cycle through various mechanisms [1, 2] and participate in close relationships in the development of some human complex diseases [3, 4] such as the Alzheimer’s disease [5] and many types of cancers [6]. Hence, identification of disease-related lncRNAs is critical to the understanding of the pathogenesis of complex diseases systematically and may further facilitate the discovery of potential drug targets. However, since biological experiments are very expensive and time-consuming, it has become a hot topic to develop effective computational models to uncover potential disease-related lncRNAs. Up to now, existing computational models for predicting potential associations between lncRNAs and diseases can be roughly classified into two major categories. Generally, in the first category of models, biological information of miRNAs, lncRNAs or diseases will be adopted to identify potential lncRNA-disease associations. For example, Chen et al. proposed a prediction model called HGLDA based on the information of miRNAs, in which, a hypergeometric distribution test was adopted to infer potential disease related lncRNAs [7]. Chen et al. proposed a KATZ measure to predict potential lncRNA-disease associations by utilizing the information of lncRNAs and diseases [8]. Ping and Wang et al. proposed a method for identifying potential disease-related lncRNAs based on the topological information of known lncRNA-disease association network [9]. In the second category of models, multiple data sources will be integrated to construct all kinds of heterogeneous networks to infer potential associations between diseases and lncRNAs. For example, Yu and Wang et al. proposed a naïve Bayesian Classifier based probability model to uncover potential disease-related lncRNAs by integrating known miRNA-disease associations, miRNA-lncRNA associations, lncRNA-disease associations, gene-lncRNA associations, gene-miRNA associations and gene-disease associations [10]. Zhang et al. developed a computational model to discover possible lncRNA-disease associations through combining lncRNAs similarity, protein-protein interactions and diseases similarity [11]. Fu et al. presented a prediction model by considering the quality and relevance of different heterogeneous data sources to identify potential lncRNA-disease associations [12]. Chen et al. proposed a novel prediction model called LRLSLDA by adopting Laplacian Regularized Least Squares to integrate known phenome-lncRNAome network, disease similarity network and lncRNA similarity network [13].

In recent years, in order to solve the problem of scarce known associations between different objects, an increasing number of recommender systems have been developed to increase the reliability of association prediction based on collaborative filtering methods [14], which depend on prior disposals to predict user-item relationships. Up to now, some novel prediction models have been proposed successively, in which, recommender algorithms have been appended to identify different potential disease-related objects. For example, Lu et.al proposed a model called SIMCLDA to predict potential lncRNA-disease associations based on inductive matrix completion by computing Gaussian interaction profile kernel of known lncRNA-disease associations, disease-gene and gene-gene onotology associations [15]. Luo et al. modeled drug repositioning problem into a recommendation system to predict novel drug indications based on known drug-disease associations through utilizing matrix completion [16]. Zeng et.al developed a novel prediction model called PCFM by adopting the probability-based collaborative filtering algorithm to infer gene-associated human diseases [17]. Luo et al. proposed a prediction model named CPTL to uncover potential disease-associated miRNAs via transduction learning by integrating disease similarity, miRNA similarity and known miRNA-disease associations [18].

In this study, a novel Collaborative Filtering model called CFNBC for predicting potential lncRNA-disease associations is proposed on the basis of Naïve Bayesian Classifier, in which, an original lncRNA-miRNA-disease tripartite network is constructed first by integrating miRNA-disease association network, miRNA-lncRNA association network and lncRNA-disease association network, and then, considering the fact that the number of known associations between the three objects such as lncRNAs, miRNAs and diseases is very limited, an updated tripartite network is further constructed by applying a collaborative filtering algorithm on the original tripartite network. Thereafter, based on the updated tripartite network, we can predict potential lncRNA-disease associations through adopting the Naïve Bayesian Classifier. Finally, in order to evaluate the prediction performance of our newly proposed model, LOOCV is implemented for CFNBC based on known experimentally verified lncRNA-disease associations. As a result, CFNBC can achieve a reliable AUC of 0.8576, which is much better than that of previous classical prediction models. Moreover, case studies of glioma, colorectal cancer and gastric cancer demonstrate the excellent prediction performance of CFNBC as well.

## Results

### Leave-one-out cross validation

In this section, in order to estimate the prediction performance of CFNBC, LOOCV will be implemented based on known experimentally verified lncRNA-disease associations. During simulation, for a given disease *d*_*j*_, each known lncRNA related to *d*_*j*_ will be left out in turns as the test sample, whereas all the remaining associations between lncRNAs and *d*_*j*_ are taken as training cases for model learning. Thus, the similarity scores between candidate lncRNAs and *d*_*j*_ can be calculated and all candidate lncRNAs can be ranked by predicted results simultaneously. As a result, the higher the candidate lncRNA is ranked, the better the performance of our prediction model will be. Moreover, the value of area under the receive operating characteristic (ROC) curve (AUC) can be further used to measure the performance of CFNBC. Obviously, the closer the AUC value is to 1, the better the prediction performance of CFNBC will be. Hence, by setting different classification thresholds, we can calculate the true positive rate (*TPR* or sensitivity) and the false positive rate (*FPR* or 1-specificity) as follows:

1 $$ TPR=\frac{TP}{TP+ FN} $$

2 $$ FPR=\frac{FP}{FP+ TN} $$

Here, *TP*, *FN*, *FP* and *TN* denote the true positives, false negatives, false positives and true negatives respectively. Specifically, *TPR* indicates the percentage of candidate lncRNAs with ranks higher than a given rank cutoff, and *FPR* denotes the percentage of candidate lncRNAs with ranks below the given threshold.

### The effects of **α**

Based on the assumption that original common neighboring miRNA nodes shall deserve more credibility than recommended common neighboring miRNA nodes, a decay factor α is used to make our prediction model CFNBC work more effectively. In this section, in order to evaluate the effects of α to the predcition performance of CFNBC, we will implement a series of experiments to estimate its actual effects while α is set to different values ranging from 0.05 to 0.8. As shown in Table 1, it is easy to see that CFNBC can achieve the best prediction performance while α is set to 0.05.

**Table 1:** The comparison results of AUCs achieved by our model by setting different values of α

α	AUCs
0.05	0.8576
0.1	0.8551
0.2	0.8482
0.3	0.8412
0.4	0.8344
0.5	0.8283
0.6	0.8228
0.7	0.8177
0.8	0.8129
0.9	0.8042

### Comparison with other state-of-the-art methods

**Fig. 1:**
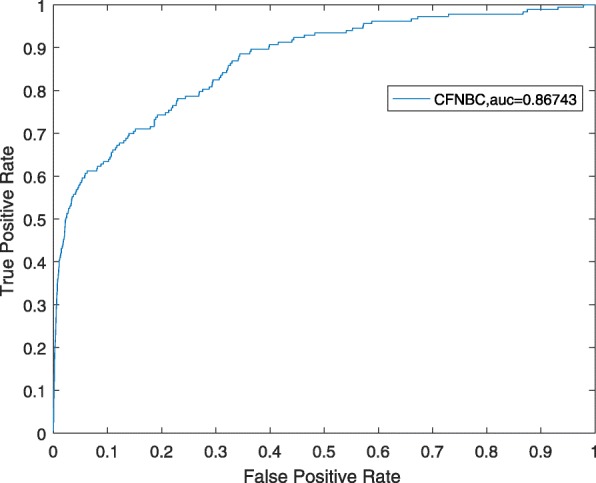
the performance of CFNBC in terms of ROC curves and AUCs based on 183 known lncRNA-disease associations under the framework of LOOCV

**Fig. 2:**
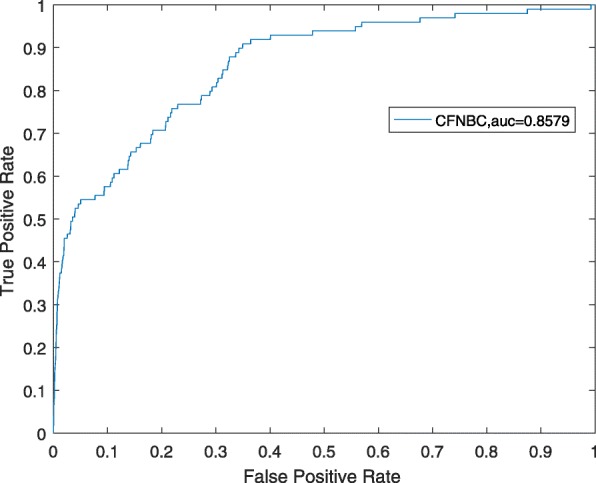
the performance of CFNBC in terms of ROC curves and AUCs based on 101 known lncRNA-disease associations under the framework of LOOCV

**Fig. 3:**
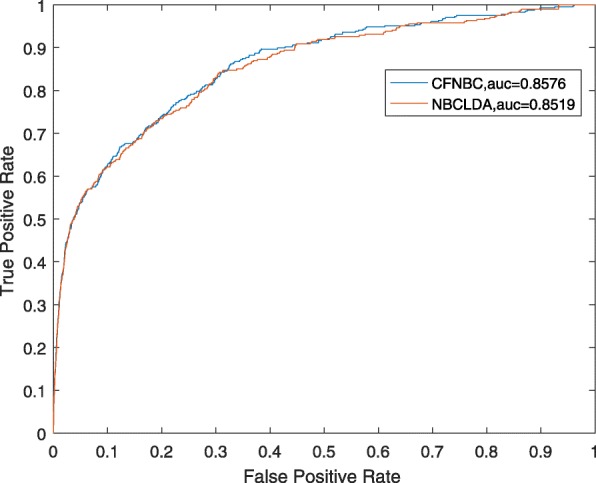
the performance of CFNBC and NBCLDA in terms of ROC curves and AUCs based on the data set *DS*_*ld*_ under the framework of LOOCV

**Fig. 4:**
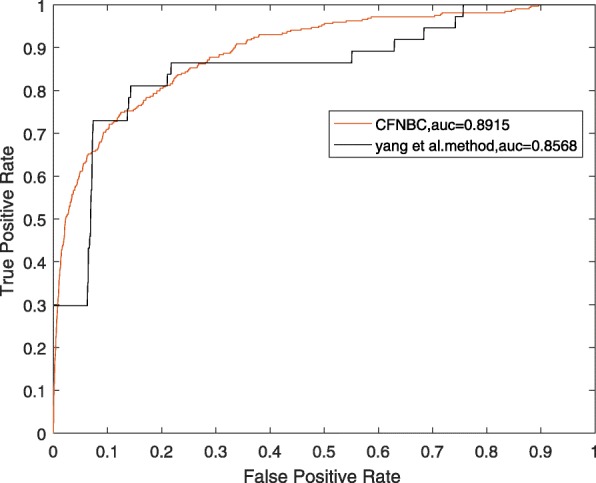
the performance of CFNBC and the method proposed by Yang et al. in terms of ROC curves and AUCs based on a data set consisting of 319 known lncRNA-disease associations under the framework of LOOCV

In order to further assess the performance of CFNBC, in this section, we will compare it with four kinds of state-of-the-art prediction models such as HGLDA [7], SIMLDA [15], NBCLDA [10] and the method proposed by Yang et al. [19] in the framework of LOOCV while α is set to 0.05. Among these four methods, since a hypergeometric distribution test was utilized to infer lncRNA-disease associations by integrating miRNA-disease associations with lncRNA-miRNA associations in HGLDA, then we will adopt a data set consisting of 183 experimentally validated lncRNA-disease associations as the hypergeometric distribution test to compare CFNBC with HGLDA. As illustrated in Table 2 and Fig. 1, the simulation results demonstrate that CFNBC outperforms HGLDA significantly. As for the model SIMLDA, since it applied inductive matrix completion to identify lncRNA-disease associations by integrating lncRNA-disease associations, gene-disease and gene-gene ontology associations, then we will collect a sub data set, which belongs to *DS*_*ld*_ in CFNBC and consists of 101 known associations between 30 different lncRNAs and 79 different diseases, from the data set adopted by SIMLDA to compare CFNBC with SIMLDA. As shown in Table 2 and Fig. 2, it is easy to see that CFNBC can achieve a reliable AUC of 0.8579, which is better than the AUC of 0.8526 achieved by SIMLDA. As for the model NBCLDA, since it fused multiple heterogeneous biological data sources and adopted the naïve Bayesian classifier to uncover potential lncRNA–disease associations, then we will compare CFNBC with it based on the data set *DS*_*ld*_ directly. As illustrated in Table 2 and Fig. 3, it is obvious that CFNBC can obtain a reliable AUC of 0.8576, which is higher than the AUC of 0.8519 achieved by NBCLDA as well. Finally, while comparing CFNBC with the method proposed by yang et al., in order to keep the fairness in comparison, we will collect a data set consisting of 319 lncRNA-disease associations between 37 lncRNAs and 52 diseases by deleting the nodes with degree equal to 1 on the data set *DS*_*ld*_. As shown in Table 2 and Fig. 4, it is easy to see that CFNBC can achieve a reliable AUC of 0.8915, which considerably outperforms the AUC of 0.8568 achieved by the method proposed by yang et al. Hence, it is easy to draw a conclusion that our model CFNBC can achieve better performance than these classical prediction models.

**Table 2:** Performance comparisons between CFNBC and some state-of-the-art models in terms of AUCs based on the different data sets of known lncRNA-disease association in the framework of the LOOCV

Methods	AUCs	Methods	AUCs
CFNBC	0.8674	CFNBC	0.8576
HGLDA	0.7621	NBCLDA	0.8519
CFNBC	0.8579	CFNBC	0.8915
SIMLDA	0.8526	Yang et al.’s method	0.8568

Additionally, in order to further evaluate the prediction performance of CFNBC, we will compare it with above four models based on the predicted top-*k* associations by using F1-score measure. During simulation, we will randomly choose 80% of known lncRNA-disease associations as the training set, whereas all remaining known and unknown lncRNA-disease associations are taken as testing sets. Since the sets of known lncRNA-disease associations in these models are different, we will set different threshold *k* to compare them with CFNBC. As shown in Table 3, it is easy to see that CFNBC outperforms these four kinds of state-of-the-art models in terms of F1-score measure as well. Moreover, the paired *t*-test also demonstrates that the performance of CFNBC is significantly better than the prediction results of other methods in terms of the F1-scores (*p*-value < 0.05, as illustrated in Table 4).

**Table 3:** F1-score of CFNBC, SIMCLDA, NBCLDA, Yang et al.’s method at different top-k cutoffs

Methods	F1-Score		
CFNBC	0.1685(k = 15)	0.1582(k = 20)	0.1422(k = 25)
SIMCLDA	0.1577(k = 15)	0.1482(k = 20)	0.1422(k = 25)
CFNBC	0.1101(k = 20)	0.1179(k = 30)	0.1079(k = 40)
NBCLDA	0.0876(k = 20)	0.0823(k = 30)	0.0875(k = 40)
CFNBC	0.2987(k = 20)	0.2778(k = 30)	0.2844(k = 40)
Yang et al.’s method	0.2678(k = 20)	0.2821(k = 30)	0.2844(k = 40)

**Table 4:** *P*-values Achieved by paired t-Test the F1-scores from top-1 to top-20 cutoffs

	SIMCLDA	NBCLDA	Yang et al.’s method
*p*-values	5.48988E-03	3.40847E-03	3.01462E-05

## Case studies

In order to further demonstrate the capability of CFNBC in inferring new lncRNAs related to a given disease, in this section, we will implement case studies of glioma, colorectal cancer and gastric cancer for CFNBC based on the data set *DS*_*ld*_. As a result, the top 20 disease-related lncRNAs predicted by CFNBC have been confirmed by manually mining relevant literatures, and corresponding evidences are listed in the following Table 5. Additionally, among these three kinds of cancers chosen for case studies, the glioma is one of the most lethal primary brain tumors with a median survival of less than 12 months, and 6 out of 100000 people may have gliomas [20], hence it is important to find potential associations between glioma and dysregulations of some lncRNAs. As illustrated in Table 5, while applying CFNBC to predict candidate lncRNAs related to glioma, it is easy to see that there are six out of the top 20 predicted glioma-related lncRNAs having been validated by recent literatures on biological experiments. For instance, the lncRNA XIST has been demonstrated to be an important regulator in tumor progression and may be a potential therapeutic target in the treatment of glioma [21]. Ma et al. found that the lncRNA MALAT1 plays an important role in glioma progression and prognosis and may be considered as a convictive prognostic biomarker for glioma patients [22]. Xue et al. provided a comprehensive analysis of KCNQ1OT1-miR-370-CCNE2 axis in human glioma cells and a novel strategy for glioma treatment [23].

**Table 5:** The lncRNAs in the top 20 for the three case studies

Diseases	lncRNAs	Evidence (PMID)	Rank
Glioma	XIST	28287613, 29187887, 28469789, 28831025	1
Glioma	MALAT1	27134488,28551849,26649728, 25613066,27904771	3
Glioma	KCNQ1OT1	28381990	5
Glioma	SNHG16	29529599	6
Glioma	NEAT1	27556696	8
Glioma	H19	29391808,26983719,29422115,27543358,27981546	19
Colorectal cancer	XIST	17143621,29495975,29137332,17143621	1
Colorectal cancer	MALAT1	21503572,27777857,27165481,26887056,25446987	3
Colorectal cancer	KCNQ1OT1	16965397,23660942,26868975	4
Colorectal cancer	NEAT1	26549670	7
Colorectal cancer	SNHG16	27693121	9
Colorectal cancer	H19	27027436,26989025,26068968	15
Gastric cancer	XIST	29053187,29212249,27911852,27620004	1
Gastric cancer	MALAT1	29162158,28942451,26871474,24857172,27887846	3
Gastric cancer	SNHG16	29081409	8
Gastric cancer	NEAT1	29363783,28401449,27095450	9
Gastric cancer	H19	29687854,27592063,26160158,28105222,29207111	13
Gastric cancer	TUG1	27983921,29719612,28927144,27261864,26913601	17

As for the colorectal cancer (CRC), it is the third most common cancer and the third leading cause of cancer death in men and women in the United States [24]. In recent years, accumulating evidences have shown that many CRC-related lncRNAs have been reported based on biological experiments. For example, Song et al. demonstrated that the higher expression of XIST was correlated with worse disease free survival of CRC patients [25]. Zheng et al. proved that the higher expression level of MALAT1 may serve as a negative prognostic marker in stage II/III CRC patients [26]. Nakano et al. found that the loss of imprinting of the lncRNA KCNQ1OT1 may play an important role in the occurrence of CRC [27]. As illustrated in Table 5, while applying CFNBC to uncover candidate lncRNAs related to CRC, it is obvious that there are 6 out of the top 20 predicted CRC-related lncRNAs having been verified in the Lnc2Cancer database.

Moreover, the gastric cancer is the second most frequent cause of cancer death [28]. Up to now, lots of lncRNAs have been reported to be associated with gastric cancer. For instance, XIST, MALAT1, SNHG16, NEAT1, H19 and TUG1 were reported to be upregulated in gastric cancer [29–34]. As illustrated in Table 5, while applying CFNBC to uncover candidate lncRNAs related to gastric cancer, it is obvious that there are 6 out of the top 20 newly identified lncRNAs related to gastric cancer having been validated by the lncRNADisease and Lnc2Cancer database respectively.

## Discussion

Accumulating evidences have shown that prediction of potential lncRNA-disease associations is helpful in understanding crucial roles of lncRNAs in biological process, complex disease diagnoses, prognoses and treatments. In this manuscript, we constructed an original lncRNA-miRNA-disease tripartite network by combining miRNA-lncRNA, miRNA-disease and lncRNA-disease associations first. And then, we formulated the prediction of potential lncRNA-disease associations as a problem of recommender system and obtained an updated tripartite network through applying a novel item-based collaborative filtering algorithm to the original tripartite network. Finally, we proposed a prediction model called CFNBC to infer potential associations between lncRNAs and diseases by applying the naïve Bayesian Classifier on the updated tripartite network. Comparing with state-of-the-art prediction models, CFNBC can achieve better performs in terms of AUC values without entirely relying on known lncRNAs-disease associations, which means that CFNBC can predict potential associations between lncRNAs and diseases even as these lncRNAs and diseases are not in known data sets. Additionally, we implemented LOOCV to evaluate the prediction performance of CFNBC, and the simulation results showed that the problem of limited positive samples existed in state-of-the-art models has been significantly solved in CFNBC by the addition of collaborative filtering algorithm and the predictive accuracy has been improved by adopting the disease semantic similarity to infer potential associations between lncRNAs and diseases. Moreover, case studies of glioma, colorectal cancer and gastric cancer were implemented to further estimate the performance of CFNBC, and simulation results demonstrated that CFNBC could be a useful tool for predicting potential relationships between lncRNAs and diseases as well. Of course, despite the reliable experimental results achieved by CFNBC, there are still some biases in our model. For example, it is noteworthy that there are many other types of data that can be utilized to uncover potential lncRNA-disease associations, therefore, the prediction performance of CFNBC would be improved by the addition of more types of data. In addition, the results of CFNBC may be affected by the quality of datasets and the numbers of known lncRNA-disease relationships as well. Furthermore, successfully established models in the other computational fields would inspire the development of lncRNA-disease association prediction, such as microRNA-disease association prediction [35–37], drug-target interaction prediction [38] and synergistic drug combinations prediction [39].

## Conclusion

Finding out lncRNA-disease relationships is essential for understanding human disease mechanisms. In this manuscript, our main contributions are as follows: (1) An original tripartite network is constructed by integrating a variety of biological information including miRNA-lncRNA, miRNA-disease and lncRNA-disease associations. (2) An updated tripartite network is constructed by applying a novel item-based collaborative filtering algorithm on the original tripartite network. (3) A novel prediction model called CFNBC is developed based on the naïve Bayesian Classifier and applied on the updated tripartite network to infer potential associations between lncRNAs and diseases. (4) CFNBC can be adopted to predict a potential disease-related lincRNA or an potential lncRNA-related disease without relying on any known lncRNA-disease associations. (5) A recommendation system is applied in CFNBC, which guarantees that CFNBC can achieve effective prediction results in condition of scarce known lncRNA-disease associations.

### Data collection and preprocessing

In order to construct our novel prediction model CFNBC, we combined three kinds of heterogeneous data sets such as the miRNA-disease association set, the miRNA-lncRNA association set and the lncRNA-disease association set to infer potential associations between lncRNAs and diseases, which were collected from different public databases including the HMDD [40], the starBase v2.0 [41], and the MNDR v2.0 databases [42], etc.

#### Construction of the miRNA-disease and miRNA-lncRNA association sets

Firstly, we downloaded two datasets of known miRNA-disease associations and miRNA-lncRNA associations from the HMDD [40] in August 2018 and the starBase v2.0 [41] in January 2015 respectively. Then, we removed duplicated associations with conflicting evidences on these two data sets separately, manually picked out the common miRNAs existing in both the dataset of miRNA-disease associations and the dataset of miRNA-lncRNA associations, and retained only the associations related with these selected miRNAs in these two data sets. As a result, we finally obtained a data set *DS*_*md*_ including 4704 different miRNA-disease interactions between 246 different miRNAs and 373 different diseases, and a data set *DS*_*ml*_ including 9086 different miRNA-lncRNA interactions between 246 different miRNAs and 1089 different lncRNAs (see Supplementary Materials Table 1and Table 2).

#### Construction of the lncRNA-disease association set

Firstly, we downloaded a dataset of known lncRNA-disease associations from the MNDR v2.0 databases [42] in 2017. Then, once the dataset was collected, in order to keep the uniformity of disease names, we transformed some diseases names included in the set of lncRNA-disease associations into their aliases in the data set of miRNA-disease associations, and unified the names of lncRNAs in the datasets of miRNA-lncRNA associations and lncRNA-diseases associations. By this means, we selected out these lncRNA-disease interactions associated with both lncRNAs belonging to *DS*_*ml*_ and diseases belonging to *DS*_*md*_. As a result, we finally obtained a data set *DS*_*ld*_ including 407 different lncRNA-disease interactions between 77 different lncRNAs and 95 different diseases (see Supplementary Materials Table 3).

#### Analysis of relational data sources

**Fig. 5:**
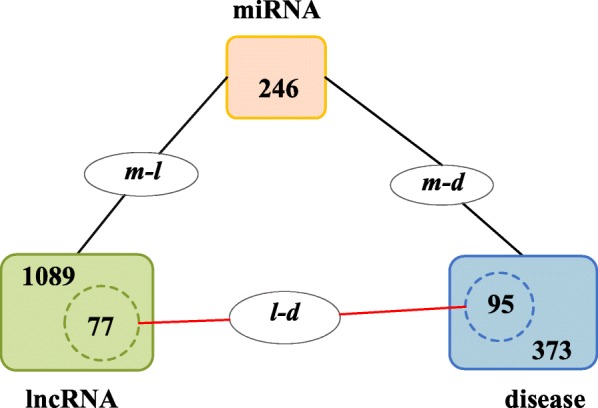
The relationships among three kinds of different data sources

In CFNBC, the newly constructed lncRNA-miRNA-disease tripartite network (LMDN for abbreviation) consists of three kinds of objects such as lncRNAs, miRNAs and diseases. Therefore, we collected three kinds of relational data sources from different databases based on these three kinds of objects. As illustrated in Fig. 5, the numbers of diseases are 373 in the data set of miRNA-disease associations (*m-d* for abbreviation) and 95 in the data set of lncRNA-disease associations (*l-d* for abbreviation) respectively. The numbers of lncRNAs are 1089 in the data set of miRNA-lncRNA associations (*m-l* for abbreviation) and 77 in *l-d* respectively. The numbers of miRNAs are 246 in both *m-l* and *m-d*. Moreover, it is clear that the set of 95 diseases in *l-d* is a subset of the set of 373 diseases in *m-d*, and the set of 77 lncRNAs in *l-d* is a subset of the set of 1089 lncRNAs in *m-l*.

## Method

**Fig. 6:**
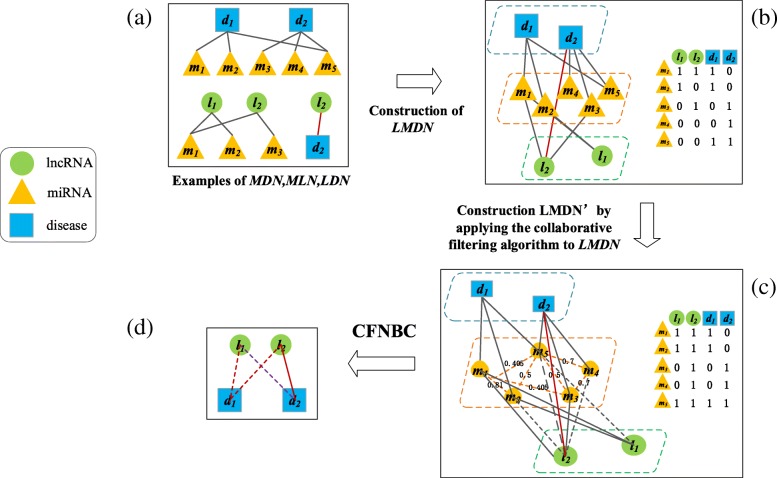
Flowchart of CFNBC. In the diagram, the green circles, blue squares, and orange triangles represent lncRNAs, diseases and miRNAs respectively. **a** construction of MDN, MLN and LDN; (**b**) construction of the original tripartite network LMDN and its corresponding adjacency matrix; (**c**) construction of the updated tripartite network LMDN^′^ and its corresponding adjacency matrix; (**d**) prediction of potential lncRNA-disease associations through applying the naïve Bayesian classifier on LMDN^′^

As illustrated in Fig. 6, our newly proposed prediction model CFNBC consists of the following four main stages:

- **Step1**: As illustrated in Fig. 6(*a*), we can construct a miRNA-disease association network MDN, a miRNA-lncRNA association network MLN, and an lncRNA-disease association network LDN based on the data sets *DS*_*md*_, *DS*_*ml*_ and *DS*_*ld*_ respectively.
- **Step2**: As illustrated in Fig. 6(*b*), through integrating these three newly constructed association networks MDN, MLN, and LDN, we can further construct an original lncRNA-miRNA-disease association tripartite network LMDN.
- **Step3**: As illustrated in Fig. 6(*c*), after applying the collaborative filtering algorithm on LMDN, we can obtain an updated lncRNA-miRNA-disease association tripartite network LMDN^′^.
- **Step4**: As illustrated in Fig. 6(*d*), after appending the naïve Bayesian classifier to LMDN^′^, we can obtain our final prediction model CFNBC.

In the original tripartite network LMDN, owing to the sparse known associations between lncRNAs and diseases, for any given lncRNA node *a* and disease node *b*, it is obvious that the number of miRNA nodes that associate with both *a* and *b* will be very limited. Hence, in CFNBC, we designed a collaborative filtering algorithm for recommending suitable miRNA nodes to corresponding lncRNA nodes and disease nodes respectively. And then, based on these known and recommended common neighboring nodes, we can finally apply the Naïve Bayesian Classifier on LMDN^′^ to uncover potential lncRNA-disease associations.

### Construction of LMDN

Let matrix ${R}_{MD}^0$ be the original adjacency matrix of known miRNA-disease associations and the entity ${R}_{MD}^0\left({m}_k,{d}_j\right)$ denote the element in the *k*^th^ row and *j*^th^ column of ${R}_{MD}^0$, then there is ${R}_{MD}^0\left({m}_k,{d}_j\right)$ =1 if and only if the miRNA node *m*_*k*_ is associated with the disease node *d*_*j*_, otherwise, there is ${R}_{MD}^0\left({m}_k,{d}_j\right)$ =0. In the same way, we can obtain the original adjacency matrix ${R}_{ML}^0$ of known miRNA-lncRNA associations as well, and in ${R}_{ML}^0$, there is ${R}_{ML}^0\left({m}_k,{l}_i\right)$ =1 if and only if the miRNA node *m*_*k*_ is associated with the lncRNA node *l*_*i*_, otherwise, there is ${R}_{ML}^0\left({m}_k,{l}_i\right)$ =0. Additionally, considering that a recommender system may involve various input data including users and items, therefore, in CFNBC, we will take lncRNAs and diseases as users, while miRNAs as items. Thereafter, as for these two original adjacency matrices ${R}_{MD}^0$ and ${R}_{ML}^0$ obtained above, since their row vectors are the same, it is easy to see that we can construct another adjacency matrix ${R}_{ML D}^0=\left[{R}_{ML}^0,{R}_{MD}^0\right]$ by splicing ${R}_{MD}^0$ and ${R}_{ML}^0$ together. Moreover, it is obvious that the row vector of ${R}_{MLD}^0$ is exactly the same as the row vector in ${R}_{MD}^0$ or ${R}_{ML}^0$, while the column vector of ${R}_{MLD}^0$ consists of the column vector of ${R}_{MD}^0$ and the column vector of ${R}_{ML}^0$.

### Applying the item-based collaborative filtering algorithm on LMDN

Since CFNBC is based on the collaborative filtering algorithm, then the relevance scores between lncRNAs and diseases predicted by CFNBC will depend on the common neighbors between these lncRNAs and diseases. However, owing to the scarce known lncRNA-miRNA, lncRNA-disease and miRNA-disease associations, the number of common neighbors between these lncRNAs and diseases in LMDN will be very limited as well. Hence, in order to improve the number of common neighbors between lncRNAs and diseases in LMDN, we will apply the collaborative filtering algorithm on LMDN in this section.

First, on the basis of $\kern0.50em {R}_{MLD}^0$ and LMDN, we can obtain a co-occurrence matrix *R*^*m* × *m*^, in which, let the entity *R*(*m*_*k*_, *m*_*r*_) denote the element in the *k*^th^ row and *r*^th^ column of *R*^*m* × *m*^, then there is *R*(*m*_*k*_, *m*_*r*_) =1 if and only if the miRNA node *m*_*k*_ and the miRNA node *m*_*r*_ share at least one common neighboring node (a lncRNA node or a disease node) in LMDN, otherwise, there is *R*(*m*_*k*_, *m*_*r*_) =0. Hence, a similarity matrix *R*^′^ can be calculated after normalizing *R*^*m* × *m*^ as follows:

3 $$ {R}^{\hbox{'}}\left({m}_k,{m}_r\right)=\frac{\mid N\left({m}_k\right)\cap N\left({m}_r\right)\mid }{\sqrt{\left|N\left({m}_k\right)\right|\ast \mid N\left({m}_r\right)\mid }}\ \left(k,r\in \left[1,246\right]\right) $$

Where ∣*N*(*m*_*k*_)∣ represents the number of known lncRNAs and diseases associated to *m*_*k*_ in LMDN, that is, the number of elements with value equaling to 1 in the *k*^th^ row of ${R}_{MLD}^0$, |*N*(*m*_*r*_)| represents the number of elements with value equaling to 1 in the *r*^th^ row of ${R}_{MLD}^0$, and ∣*N*(*m*_*k*_) ∩ *N*(*m*_*r*_)∣ denotes the number of known lncRNAs and diseases associated with both *m*_*k*_ and *m*_*r*_ simultaneously in LMDN.

Next, for any given lncRNA node *l*_*i*_ and miRNA node *m*_*h*_ in LMDN, if the association between *l*_*i*_ and *m*_*h*_ is known already, then, for a miRNA node *m*_*t*_ other than *m*_*h*_ in LMDN, it is obvious that the higher the relevance score between *m*_*t*_ and *m*_*h*_, the bigger the possibility that there may exist potential association between *l*_*i*_ and *m*_*t*_. Hence, we can obtain the relevance score between *l*_*i*_ and *m*_*t*_ based on the similarities between miRNAs as follows:

4 $$ {p}_{l_i{m}_t=\sum \limits_{m_t\in N\left({l}_i\right)\cap S\left(K,{m}_t- top\right)}{R}_t^{\prime}\times {u}_{it}} $$

Here, *N*(*l*_*i*_) represents the set of neighboring miRNA nodes that are directly connected to *l*_*i*_ in LMDN, and *S*(*K*, *m*_*t*_ − *top*) denote the set of top-*K* miRNAs that are most similar to *m*_*t*_ in LMDN. ${R}_t^{\prime }$ is a vector consisting of the *t*^th^ row of *R*^′^. In addition, there is *u*_*it*_ = 1 if and only if *l*_*i*_ is interacted with *m*_*t*_ in ML, otherwise, there is *u*_*it*_ =0.

Similarly, for any given disese node *d*_*j*_ and miRNA node *m*_*h*_ in LMDN, if the association between *d*_*j*_ and *m*_*h*_ is known already, then, for a miRNA node *m*_*t*_ other than *m*_*h*_ in LMDN, we can obtain the relevance score between *d*_*j*_ and *m*_*t*_ based on the similarities between miRNAs as follows:

5 $$ {p}_{d_j{m}_t=\sum \limits_{m_t\in N\left({d}_j\right)\cap S\left(K,{m}_t- top\right)}{R}_t^{\prime}\times {u}_{jt}} $$

Where *N*(*d*_*j*_) denotes the set of neighboring miRNA nodes that are directly connected to *d*_*j*_ in LMDN. In addition, there is *u*_*jt*_ =1 if and only if *d*_*j*_ is interacted with *m*_*t*_ in MD, otherwise, there is *u*_*jt*_ =0.

Obviously, based on the similarity matrix *R*^′^ and the adjacency matrix ${R}_{MLD}^0$, we can construct a new recommender matrix ${R}_{MLD}^1$ as follows:

6 $$ {R}_{MLD}^1={R}^{\prime}\times {R}_{MLD}^0 $$

In particular, for a certain lncRNA node *l*_*i*_ or a disease node *d*_*j*_ in LMDN, if there is a miRNA *m*_*k*_ satisfying ${R}_{MLD}^0\left({m}_k,{l}_i\right)=1$ or ${R}_{MLD}^0\left({m}_k,{d}_j\right)=1$ in ${R}_{MLD}^0$, then, we will first sum up the values of all elements in the *i*^th^ or *j*^th^ column of ${R}_{MLD}^1$ respectively. Thereafter, we will obtain its average value $\overline{p}$. Finally, if there is a miRNA node *m*_*θ*_ in the *i*^th^ or *j*^th^ column of ${R}_{MLD}^1$ satisfying ${R}_{MLD}^1\left({m}_{\theta },{l}_i\right)>\overline{p}$ or ${R}_{MLD}^1\left({m}_{\theta },{d}_j\right)>\overline{p}$, then we will recommend the miRNA *m*_*θ*_ to *l*_*i*_ or *d*_*j*_ respectively. And in the same time, we will as well add a new edge between *m*_*θ*_ and *l*_*i*_ or *m*_*θ*_ and *d*_*j*_ in LMDN separately.

For instance, according to Fig. 6 and the given matrix $$ {R}_{MLD}^0=\left[\begin{array}{cc}\begin{array}{cc}1& 1\\ {}1& 0\end{array}& \begin{array}{cc}1& 0\\ {}1& 0\end{array}\\ {}\begin{array}{cc}0& 1\\ {}\begin{array}{c}0\\ {}0\end{array}& \begin{array}{c}0\\ {}0\end{array}\end{array}& \begin{array}{cc}0& 1\\ {}\begin{array}{c}0\\ {}1\end{array}& \begin{array}{c}1\\ {}1\end{array}\end{array}\end{array}\right] $$, we can obtain its corresponding matrices *R*^*m* × *m*^, *R*^′^ and ${R}_{MLD}^1$ as follows:

7 $$ {R}^{m\times m}=\left[\begin{array}{ccccc}\backslash & 1& 1& 0& 1\\ {}1& \backslash & 0& 0& 1\\ {}1& 0& \backslash & 1& 1\\ {}0& 0& 1& \backslash & 1\\ {}1& 1& 1& 1& \backslash \end{array}\right] $$

8 $$ {R}_{MLD}^1=\left[\begin{array}{ccccc}\backslash & 0.81& 0.405& 0& 0.405\\ {}0.81& \backslash & 0& 0& 0.5\\ {}0.405& 0& \backslash & 0.7& 0.5\\ {}0& 0& 0.7& \backslash & 0.7\\ {}0.405& 0.5& 0.5& 0.7& \backslash \end{array}\right] $$

9 $$ {R}_{MLD}^1=\left[\begin{array}{cccc}0.81& 0.405& 1.215& 0.81\\ {}0.81& 0.81& 1.31& 0.5\\ {}0.405& 0.405& 0.905& 1.2\\ {}0& 0.7& 0.7& 1.4\\ {}0.905& 0.905& 0.905& 1.2\end{array}\right] $$

To be specific, as illustrate in Figure 6, if taking the lncRNA node *l*_1_ as an example, then from the matrix ${R}_{MLD}^0$, it is easy to see that there are two miRNA nodes such as *m*_1_ and *m*_2_ associated with *l*_1_. In addition, according to formula (9), we can know as well that there is ${R}_{MLD}^1\left({m}_5,{l}_1\right)=0.905>\overline{p}=\frac{R_{MLD}^1\left({m}_1,{l}_1\right)+{R}_{MLD}^1\left({m}_2,{l}_1\right)}{2}=\frac{0.81+0.81}{2}=0.81$. Hence, we will recommend the miRNA node *m*_5_ to *l*_1_. In the same way, the miRNA nodes *m*_2_, *m*_4_ and *m*_5_ will be recommended to *l*_2_ as well. Moreover, according to previous description, it is obvious that these new edges between *m*_5_ and *l*_1_, *m*_2_ and *l*_2_, *m*_4_ and *l*_2_, and *m*_5_ and *l*_2_ will be added to the original tripartite network LMDN in the same time. Thereafter, we can obtain an updated lncRNA-miRNA-disease association tripartite network LMDN^′^ on the basis of the original tripartite network LMDN.

### Construction of the prediction model CFNBC

The naïve Bayesian classifier is a kind of simple probabilistic classifier with a conditionally independent assumption. Based on this probability model, the posterior probability can be described as follows:

10 $$ p\left(C|{F}_1,{F}_2,\cdots, {F}_n\right)=\frac{p\left({F}_1,{F}_2,\cdots, {F}_n|C\right)p(C)}{p\left({F}_1,{F}_2,\cdots, {F}_n\right)} $$

Where *C* is a dependent class variable and *F*_1_, *F*_2_, …, *F*_*n*_ are the feature variables of class *C*.

Moreover, since each feature *F*_*i*_ is conditionally independent to any other feature *F*_*j*_ (*i* ≠ *j*) in class C, then the above formula (10) can as well be expressed as follows:

11 $$ p\left(C|{F}_1,{F}_2,\cdots, {F}_n\right)=\frac{p(C)\prod \limits_{i=1}^np\left({F}_i|C\right)}{p\left({F}_1,{F}_2,\cdots, {F}_n\right)} $$

**Fig. 7:**
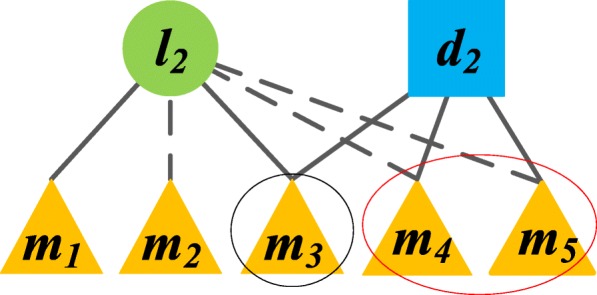
a subnetwork of Figure 6(d), in which, a solid line between a lcnRNA (or disease) node and a miRNA node means that there is a known association between these two nodes, while a dotted line between a lcnRNA (or disease) node and a miRNA node means that the association between these two nodes is obtained by our item-based collaborative filtering algorithm, then, it is easy to know that the common neighboring node *m*_3_ is an original common neighboring miRNA node of *l*_2_ and *d*_2_, while *m*_4_, *m*_5_ are recommended common neighboring miRNA nodes of *l*_2_ and *d*_2_

In our previous work, we proposed a probability model called NBCLDA based on the Naïve Bayesian classifier to predict potential lncRNA-disease associations [10]. However, in NBCLDA, there exist some circumstances where it happens to be no relevance scores between a certain pair of lncRNA and disease nodes, and the reason is that there are no common neighbors between them owing to the scarce known associations between the pair of lncRNA and disease. Hence, in order to overcome this kind of drawback existing in our previous work, in this section, we will design a novel prediction model called CFNBC to infer potential associations between lncRNAs and diseases through adopting the item-based collaborative filtering algorithm on LMDN and applying the Naïve Bayesian classifier on LMDN^′^. In CFNBC, for a given pair of lncRNA and disease nodes, it is obvious that they will have two kinds of common neighboring miRNA nodes such as the original common miRNA nodes and the recommended common miRNA nodes. In order to illustrate this case more intuitively, an example is given in Figure 7, in which, the node *m*_3_ is an original common neighboring miRNA node since it has known associations with both *l*_2_ and *d*_2_, while the nodes *m*_4_ and *m*_5_ belong to recommended common neighboring miRNA nodes since they do not have known associations with both *l*_2_ and *d*_2_. And in particular, while applying the Naïve Bayesian classifier on LMDN^′^, for a given pair of lncRNA and disease nodes, we will consider that their common neighboring miRNA nodes, including both the original and recommended common neighboring miRNA nodes, are all conditionally independent of each other, since they are different nodes in LMDN^′^. That is, for a given pair of lncRNA and disease nodes, it is assumed that all their common neighboring nodes will not interfere with each other in CFNBC.

### Method for applying the Naïve Bayesian theory on **LMDN**^′^

For any given lncRNA node *l*_*i*_ and disease node *d*_*j*_ in LMDN^′^, let *CN*_1_(*l*_*i*_, *d*_*j*_) = {*m*_1 − 1_, *m*_2 − 1_, ⋯*m*_*h* − 1_} denote a set consisting of all original common neighboring nodes between them, and *CN*_2_(*l*_*i*_, *d*_*j*_) = {*m*_1 − 2_, *m*_2 − 2_, ⋯*m*_*h* − 2_} denote a set consisting of all recommended common neighboring nodes between them in LMDN^′^, then, the prior probabilities $p\left({e}_{l_i-{d}_j}=1\right)$ and $p\left({e}_{l_i-{d}_j}=0\right)$ can be calculated as follows:

12 $$ p\left({e}_{l_i-{d}_j}=1\right)=\frac{\left|{M}^c\right|}{\left|M\right|} $$

13 $$ p\left({e}_{l_i-{d}_j}=0\right)=1-p\left({e}_{l_i-{d}_j}=1\right) $$

Where |*M*^*c*^| denotes the number of known lncRNA-disease associations in LDN and |*M*| = *nl* × *nd*. Here, *nl* and *nd* represent the number of different lncRNAs and diseases in LDN respectively.

Furthermore, based on these two kinds of common neighboring nodes, the posterior probabilities between *l*_*i*_ and *d*_*j*_ can be calculated as follows:

14 $$ p\left({e}_{l_i-{d}_j}=1|{CN}_1\left({l}_i,{d}_j\right),{CN}_2\left({l}_i,{d}_j\right)\right)=\frac{p\left({e}_{l_i-{d}_j}=1\right)}{p\left({CN}_1\Big({l}_i,{d}_j\right),{CN}_2\left({l}_i,{d}_j\right)\Big)}\prod \limits_{m_{\updelta -1}\in {CN}_1\left({l}_i,{d}_j\right)}p\left({m}_{\updelta -2}|{e}_{l_i-{d}_j}=1\right)\times \prod \limits_{m_{\updelta -2}\in {CN}_2\left({l}_i,{d}_j\right)}p\left({m}_{\updelta -2}|{e}_{l_i-{d}_j}=1\right) $$

15 $$ p\left({e}_{l_i-{d}_j}=0|{CN}_1\left({l}_i,{d}_j\right),{CN}_2\left({l}_i,{d}_j\right)\right)=\frac{p\left({e}_{l_i-{d}_j}=0\right)}{p\left({CN}_1\Big({l}_i,{d}_j\right),{CN}_2\left({l}_i,{d}_j\right)\Big)}\prod \limits_{m_{\updelta -1}\in {CN}_1\left({l}_i,{d}_j\right)}p\left({m}_{\updelta -2}|{e}_{l_i-{d}_j}=0\right)\times \prod \limits_{m_{\updelta -2}\in {CN}_2\left({l}_i,{d}_j\right)}p\left({m}_{\updelta -2}|{e}_{l_i-{d}_j}=0\right) $$

Obviously, comparing formula (14) with formula (15), it can be easily identified that whether an lncRNA node is related to a disease node or not in LMDN^′^. However, since it is too difficult to obtain the value of *p*(*CN*_1_(*l*_*i*_, *d*_*j*_)) and *p*(*CN*_2_(*l*_*i*_, *d*_*j*_)) directly, the probability of potential association existing between *l*_*i*_ and *d*_*j*_ in LMDN^′^ can be defined as follows:

16 $$ S\left({l}_i,{d}_j\right)=\frac{p\left({e}_{l_i-{d}_j}=1|{CN}_1\left({l}_i,{d}_j\right),{CN}_2\left({l}_i,{d}_j\right)\right)}{\ p\left({e}_{l_i-{d}_j}=0|{CN}_1\left({l}_i,{d}_j\right),{CN}_2\left({l}_i,{d}_j\right)\right)}=\frac{p\left({e}_{l_i-{d}_j}=1\right)}{p\left({e}_{l_i-{d}_j}=0\right)}\prod \limits_{m_{\updelta -1}\in {CN}_1\left({l}_i,{d}_j\right)}\frac{p\left({m}_{\updelta -1}|{e}_{l_i-{d}_j}=1\right)}{p\left({m}_{\updelta -1}|{e}_{l_i-{d}_j}=0\right)}\prod \limits_{m_{\updelta -2}\in {CN}_2\left({l}_i,{d}_j\right)}\frac{p\left({m}_{\updelta -2}|{e}_{l_i-{d}_j}=1\right)}{p\left({m}_{\updelta -2}|{e}_{l_i-{d}_j}=0\right)} $$

Here $p\left({m}_{\updelta -1}|{e}_{l_i-{d}_j}=1\right)$ and $p\left({m}_{\updelta -1}|{e}_{l_i-{d}_j}=0\right)$ denote the conditional possibilities that whether the node *m*_δ − 1_ is a common neighboring node between *l*_*i*_ and *d*_*j*_ or not in LMDN^′^ separately, and $p\left({m}_{\updelta -2}|{e}_{l_i-{d}_j}=1\right)$ and $p\left({m}_{\updelta -2}|{e}_{l_i-{d}_j}=0\right)$ represent whether the node *m*_δ − 2_ is a common neighboring node between *l*_*i*_ and *d*_*j*_ or not in LMDN^′^ respectively. Moreover, according to the Bayesian theory, these four kinds of conditional probabilities can be defined as follows:

17 $$ p\left({m}_{\updelta -1}|{e}_{l_i-{d}_j}=1\right)=\frac{p\left({e}_{l_i-{d}_j}=1|{m}_{\updelta -1}\right)p\left({m}_{\updelta -1}\right)}{p\left({e}_{l_i-{d}_j}=1\right)} $$

18 $$ p\left({m}_{\updelta -1}|{e}_{l_i-{d}_j}=0\right)=\frac{p\left({e}_{l_i-{d}_j}=0|{m}_{\updelta -1}\right)p\left({m}_{\updelta -1}\right)}{p\left({e}_{l_i-{d}_j}=0\right)} $$

19 $$ p\left({m}_{\updelta -2}|{e}_{l_i-{d}_j}=1\right)=\frac{p\left({e}_{l_i-{d}_j}=1|{m}_{\updelta -1}\right)p\left({m}_{\updelta -2}\right)}{p\left({e}_{l_i-{d}_j}=1\right)} $$

20 $$ p\left({m}_{\updelta -2}|{e}_{l_i-{d}_j}=0\right)=\frac{p\left({e}_{l_i-{d}_j}=0|{m}_{\updelta -2}\right)p\left({m}_{\updelta -2}\right)}{p\left({e}_{l_i-{d}_j}=0\right)} $$

Where $p\left({e}_{l_i-{d}_j}=1|{m}_{\updelta -1}\right)$ and $p\left({e}_{l_i-{d}_j}=0|{m}_{\updelta -1}\right)$ are the probability of whether the lncRNA node *l*_*i*_ is connected to the disease node *d*_*j*_ or not respectively, while *m*_δ − 1_ is a common neighboring miRNA node between *l*_*i*_ and *d*_*j*_ in LMDN^′^. And similarly, $p\left({e}_{l_i-{d}_j}=1|{m}_{\updelta -2}\right)$ and $p\left({e}_{l_i-{d}_j}=0|{m}_{\updelta -2}\right)$ represent the probability of whether the lncRNA node *l*_*i*_ is connected to the disease node *d*_*j*_ or not respectively, while *m*_δ − 2_ is a common neighboring miRNA node between *l*_*i*_ and *d*_*j*_ in LMDN^′^. Moreover, supposing that *m*_δ − 1_ and *m*_δ − 2_ are two common neighboring miRNA nodes between *l*_*i*_ and *d*_*j*_ in LMDN^′^, let ${N}_{m_{\updelta -1}}^{+}$ and ${N}_{m_{\updelta -1}}^{-}$ represent the number of known associations and the number of unknown associations between disease nodes and lncRNA nodes in LMDN^′^ that have *m*_δ − 1_ as a common neighboring miRNA node between them, and ${N}_{m_{\updelta -2}}^{+}$ and ${N}_{m_{\updelta -2}}^{-}$ represent the number of known associations and the number of unknown associations between disease nodes and lncRNA nodes in LMDN^′^ that have *m*_δ − 2_ as a common neighboring miRNA node between them, then, it is obvious that $p\left({e}_{l_i-{d}_j}=1|{m}_{\updelta -1}\right)$ and $p\left({e}_{l_i-{d}_j}=1|{m}_{\updelta -2}\right)$ can be calculated as follows:

21 $$ p\left({e}_{l_i-{d}_j}=1|{m}_{\updelta -1}\right)=\frac{N_{m_{\updelta -1}}^{+}}{N_{m_{\updelta -1}}^{+}+{N}_{m_{\updelta -1}}^{-}} $$

22 $$ p\left({e}_{l_i-{d}_j}=1|{m}_{\updelta -2}\right)=\frac{N_{m_{\updelta -2}}^{+}}{N_{m_{\updelta -2}}^{+}+{N}_{m_{\updelta -2}}^{-}} $$

Obviously, according to above formula (17), formula (18), formula (19) and formula (20), the formula (16) can be modified as follows:

23 $$ S\left({l}_i,{d}_j\right)=\frac{p\left({e}_{l_i-{d}_j}=1\right)}{p\left({e}_{l_i-{d}_j}=0\right)}\prod \limits_{m_{\updelta -1}\in {CN}_1\left({l}_i,{d}_j\right)}\frac{p\left({e}_{l_i-{d}_j}=0\right)p\left({e}_{l_i-{d}_j}=1|{m}_{\updelta -1}\right)}{p\left({e}_{l_i-{d}_j}=1\right)p\left({e}_{l_i-{d}_j}=0|{m}_{\updelta -1}\right)}\prod \limits_{m_{\updelta -2}\in {CN}_2\left({l}_i,{d}_j\right)}\frac{p\left({e}_{l_i-{d}_j}=0\right)p\left({e}_{l_i-{d}_j}=1|{m}_{\updelta}\right)}{p\left({e}_{l_i-{d}_j}=1\right)p\left({e}_{l_i-{d}_j}=0|{m}_{\updelta}\right)} $$

Furthermore, for any given lncRNA node *l*_*i*_ and disease node *d*_*j*_, since the value of $\frac{p\left({e}_{l_i-{d}_j}=1\right)}{p\left({e}_{l_i-{d}_j}=0\right)}$ is a constant, then for convenience, we will denote the value of $\frac{p\left({e}_{l_i-{d}_j}=1\right)}{p\left({e}_{l_i-{d}_j}=0\right)}$ as *ϕ*_*m*_. In addition, for each common neighboring node *m*_δ − 1_ between *l*_*i*_ and *d*_*j*_, let *N*_*l* − 1_ and *N*_*d* − 1_ denote the numbers of lncRNAs and diseases associated to *m*_δ − 1_ in LMDN^′^ respectively, then it is obvious that there is ${N}_{m_{\updelta -1}}^{+}+{N}_{m_{\updelta -1}}^{-}={N}_{l-1}\times {N}_{d-1}$. And similarly, for each common neighboring miRNA node *m*_δ − 2_ between *l*_*i*_ and *d*_*j*_, let *N*_*l* − 2_ and *N*_*d* − 2_ represent the numbers of lncRNAs and diseases associated to *m*_δ − 2_ in LMDN^′^ respectively, then it is obvious that there is ${N}_{m_{\updelta -2}}^{+}+{N}_{m_{\updelta -2}}^{-}={N}_{l-2}\times {N}_{d-2}$. Thereafter, the above formula (16) can be further modified as follows:

24 $$ S\left({l}_i,{d}_j\right)={\phi}_m\prod \limits_{m_{\updelta -1}\in {CN}_1\left({l}_i,{d}_j\right)}\prod \limits_{m_{\updelta -2}\in {CN}_2\left({l}_i,{d}_j\right)}{\phi_m}^{-2}\frac{N_{m_{\updelta -1}}^{+}}{N_{m_{\updelta -1}}^{-}}\frac{N_{m_{\updelta -2}}^{+}}{N_{m_{\updelta}-2}^{-}} $$

Besides, since ${N}_{m_{\updelta -1}}^{+}$ and ${N}_{m_{\updelta -2}}^{+}$ may be zero, then we introduce the Laplace calibration to guarantee that the value of *S*(*l*_*i*_, *d*_*j*_) will not be zero. Hence, the above formula (16) can once again be modified as follows:

25 $$ S\left({l}_i,{d}_j\right)={\phi}_m\prod \limits_{m_{\updelta -1}\in {CN}_1\left({l}_i,{d}_j\right)}\prod \limits_{m_{\updelta -2}\in {CN}_2\left({l}_i,{d}_j\right)}{\phi_m}^{-2}\frac{N_{m_{\updelta -1}}^{+}+1}{N_{m_{\updelta -1}}^{-}+1}\frac{N_{m_{\updelta -2}}^{+}+1}{N_{m_{\updelta}-2}^{-}+1} $$

Next, for any given lncRNA node and disease node, since the original common neighboring miRNA nodes between them are obtained from the known associations, while the recommended common neighboring miRNA nodes between them are obtained by our item-based collaborative filtering algorithm, then it is reasonable to consider that the original common neighboring miRNA nodes shall deserve more credibility than the recommended common neighboring miRNA nodes. Hence, in order to make our prediction model be able to work more effectively, we will add a decay factor α in the range of (0, 1) to the above formula (25). Thereafter, the formula (25) can be rewritten as follows:

26 $$ S\left({l}_i,{d}_j\right)={\phi}_m\prod \limits_{m_{\updelta -1}\in {CN}_1\left({l}_i,{d}_j\right)}\prod \limits_{m_{\updelta -2}\in {CN}_2\left({l}_i,{d}_j\right)}{\phi_m}^{-2}\frac{N_{m_{\updelta -1}}^{+}+1}{N_{m_{\updelta -1}}^{-}+1}{\left(\frac{N_{m_{\updelta -2}}^{+}+1}{N_{m_{\updelta}-2}^{-}+1}\right)}^{\upalpha} $$

Additionally, it has been reported that the degree of common neighboring nodes will play a significant role in the link prediction, and the common neighboring nodes with high degrees can improve the prediction accuracy [43]. Hence, we will further add an index Resource (RA) [44] and Logarithmic function for standardization to the above formula (26). Thereafter, for any given lncRNA node *l*_*i*_ and disease node *d*_*j*_ in LMDN^′^, we can obtain the probability that there may exist a potential association between them as follows:

27 $$ S^{\prime}\left({l}_i,{d}_j\right)=\frac{\log S\left({l}_i,{d}_j\right)}{k_{m_{\delta -1}}{k}_{m_{\delta -2}}} $$

Here, ${k}_{m_{\delta -1}}$ and ${k}_{m_{\delta -2}}$ represent the degree of *m*_δ − 1_ and *m*_δ − 2_ in LMDN^′^ respectively.

### Method for appending the disease semantic similarity into CFNBC

Each disease can be described as a Directed Acyclic Graph (*DAG*), in which, the nodes represent the disease MeSH descriptors and all MeSH descriptors in the *DAG* are linked from parent nodes to child nodes by a direct edge. By this way, a disease *d*_*j*_ can be denoted as *DAG*(*d*_*j*_) = (*d*_*j*_, *T*(*d*_*j*_), *E*(*d*_*j*_)), where *T*(*d*_*j*_) is the set consisting of node *d*_*j*_ and its ancestor nodes, *E*(*d*_*j*_) represents the set of edges between parent nodes and child nodes [45]. Thereafter, by adopting the scheme of *DAG*, we can define the semantic value of *d*_*j*_ as follows:

28 $$ DV\left({d}_j\right)={\sum}_{t\in {T}_{d_j}}{D}_{d_j}(t) $$

Where,

29 $$ {D}_{d_j}(t)=\left\{\begin{array}{c}1\  if\ t\ne {d}_j\\ {}{D}_{d_j}(t)=\max \left\{\delta \times {D}_{d_j}(ct)| ct\in children\ of\ t\right\}\  if\ t\ne {d}_j\ \end{array}\right. $$

Here, *δ* is the semantic contribution factor with the value between 0 and 1, and according to previous work, *δ* will be set to 0.5 in this paper. Thus, based on above formula (28) and formula (29), the semantic similarity between diseases *d*_*j*_ and *d*_*i*_ can be calculated as follows:

30 $$ SD\left({d}_j,{d}_i\right)=\frac{\sum \limits_{t\in {T}_{d_j}\cap {T}_{d_i}}\left({D}_{d_j}(t)+{D}_{d_i}(t)\right)}{DV\left({d}_j\right)+ DV\left({d}_i\right)} $$

Based on above formula (25) and formula (30), for any given lncRNA node *l*_*i*_ and disease node *d*_*j*_ in LMDN^′^, we can finally obtain the probability that there may exist a potential association between them as follows:

31 $$ S=S^{\prime}\times SD $$

## Data Availability

The Matlab code can be download at https://github.com/jingwenyu18/CFNBC; The datasets generated and/or analysed during the current study are available in the HMDD repository, http://www.cuilab.cn/; MNDR repository, http://www.rna-society.org/mndr/; starBase repository, http://starbase.sysu.edu.cn/starbase2/index.php .

## Abbreviations

AUC: areas under ROC curve
CFNBC: a novel Collaborative Filtering algorithm for sparse known lncRNA-disease associations will be proposed on the basis of Naïve Bayesian Classifier
CRC: the Colorectal cancer
FPR: false positive rates
*l-d*: the data set of lncRNA-disease associations
LMDN: the lncRNA-miRNA-disease tripartite network
LMDN′: an updated lncRNA-miRNA-disease association tripartite network
lncRNA: long non-coding RNAs lncRNA
lncRNAs: long non-coding RNAs lncRNAs
LOOCV: Leave-One Out Cross Validation
*m-d*: the data set of miRNA-disease associations
*m-l*: the data set of miRNA-lncRNA associations
TPR: true positive rates
